# *JACC: Basic to Translational Science* 2016-2025: Ubi Fuisti?

**DOI:** 10.1016/j.jacbts.2025.101325

**Published:** 2025-07-28

**Authors:** Douglas L. Mann

For the second issue of the journal*,* I wrote an editorial entitled: “*JACC: Basic to Tranlstional Science*: Quo Vadis (where are you going).”^1^ For this, my final Editor’s Page as Editor-in-Chief, I wanted to look back on where *JACC: Basic to Tranlstional Science* has been (*ubi fuisti*), and to recognize and thank the many individuals whose expertise, insight, and commitment have made the first decade of the journal possible.

The guiding editorial ethos for *JACC: Basic to Tranlstional Science*, beginning with the first issue, has been to serve as “platform for accelerating the translation of new scientific discoveries into new therapies that improve clinical outcomes for patients afflicted with or at risk for cardiovascular disease.”^2^ Peter Libby coined the term *translational vector to* describe the scientific emphasis of the papers that we sought to publish. A second mission was to provide an open access forum and learning center for teams of basic and clinical investigators across academia and industry who were interested in translational research.^2^ As I reflect back on the broad array of different papers that we have published over the last 10 years and the multiple collaborations with the Cardiovascular Research Foundation and the simultaneous publications from their annual Shark Tank presentations at Technology and Cardiovascular Therapeutics and Technology and Heart Failure Therapeutics, I believe that we have remained true to our founding editorial principals.

Acquiring the quality of epistemic humility as an Editor-in-Chief is essential to establish a journalistic culture that fosters open inquiry, critical evaluation, and intellectual integrity. As I learned quickly during my first year, to be epistemically humble requires recognizing the limits of one’s own knowledge and to be receptive to diverse perspectives, even when they challenge established norms (especially my own). To this end, I have been fortunate to receive the input and guidance from an outstanding editorial board, including Peter Libby, Brian Annex and Dan Kelly, who have been on the board for 10 years; Geoff Pitt, who joined the board 9 years ago; and the other current members of the board including Coleen McNamara (Deputy Editor) and (in alphabetical order) Nick Frangogiannis, Maria Kontaridis, Robb MacLellan, and Karin Sipido. I am extremely grateful for their support and unwavering commitment to journalistic excellence. I would be remiss if I did not acknowledge the assistance of Michael Bristow, who served as Guest Editor-in-Chief for all papers that represented conflicts for the editorial board. I would also like to thank Juan Granada and Marlene Rabinovitch who were, respectively, Section Editors for papers related to cardiac devices and pediatric cardiology, as well as Ankit Garg (Scientific Graphics Editor), who literally makes us look good; Cindy Greene (Statistical Editor), who always found a way to make our papers better; and our social media editors, Matt Alexander and Jeff Hsu, who helped to spread the word.

A key measure of the success of any journal is the ability to attract outstanding scientists to assume editorial leadership. To this end, I am delighted that Dr Matthias Nahrendorf has assumed the role as Editor-in-Chief of *JACC: Basic to Tranlstional Science*, beginning with the August 2025 issue. Matthias is a brilliant scientist whose work I have followed closely and admired deeply for the past 15 years. Although I suspect he will envision the concept of a translational vector from a slightly different angle, I know that he and his Editorial Board will uphold the principals of scientific rigor and integrity that are the *sine qua non* of translational research. I am honored that he will assume the leadership of *JACC: Basic to Tranlstional Science* this August.

The enormous joy and satisfaction of launching a journal and being able to watch it grow and become part of the fabric of the scientific cardiovascular community is only matched in intensity by the complexity of emotions that I feel as I step away from something that I have loved doing and that has been an integral part of my life for the past 10 years. Among the many things that I will miss beginning this August, aside from not working with the outstanding Editorial Board, will be not being able to continue to work with the incredible Managing Editor for the journal, Kim Trevey, as well as interacting with Monica Payne Emerson (*JACC* Executive Managing Editor), Justine Turco (ACC Divisional Vice President of Publishing), and Nancy Axelrod (Executive Publishing Editor for Elsevier), who have always advocated for the mission of the journal. Most of all I will miss all of you, the many readers and authors of *JACC: Basic to Translational Science*, who have supported this endeavor for the past decade.
